# Intravenous Administration of Stromal Vascular Fraction Attenuates Early Busulfan-Induced Testicular Injury in Rats: An Alternative Cell-Based Approach for Spermatogenic Impairment

**DOI:** 10.3390/biology15151312

**Published:** 2026-08-05

**Authors:** Tiantian Li, Zhuojie Liu, Feng Yang, Linshu Ding, Qiuju Yuan, Kwok-Fai So, Yan Zhang, Wutian Wu

**Affiliations:** 1 Guangdong-Hong Kong-Macau Institute of CNS Regeneration, Ministry of Education CNS Regeneration Collaborative Joint Laboratory, Jinan University, Guangzhou 510632, China; ltt525212022@163.com (T.L.); y1072567501@126.com (F.Y.); dinglinshu@jnu.edu.cn (L.D.); qiujuyuan@gmail.com (Q.Y.); hrmaskf@hku.hk (K.-F.S.); 2 Department of Infertility and Sexual Medicine, The Third Affiliated Hospital, Sun Yat-Sen University, 600 Tianhe Road, Guangzhou 510630, China; liuzhuojie311@foxmail.com; 3 Re-Stem Biotechnology Co., Ltd., Suzhou 215129, China

**Keywords:** stromal vascular fraction, busulfan, testicular injury, intravenous injection, spermatogenesis, rat model

## Abstract

Stromal vascular fraction (SVF), an uncultured adipose-derived cell population, exerts regenerative effects primarily through paracrine mechanisms. While local intratesticular injection of SVF alleviates busulfan-induced testicular injury, the intravenous route remains unexplored. This study investigated whether intravenous SVF administration could mitigate busulfan-induced spermatogenic impairment. Adult male Sprague-Dawley rats received busulfan (15 mg/kg, intraperitoneal) or vehicle; the busulfan + SVF group received 2 × 10^6^ allogeneic SVF cells intravenously immediately after busulfan injection. Two weeks later, we evaluated sperm parameters, testicular histopathology (Johnsen’s score, seminiferous tubule diameter, germinal epithelial thickness), and immunohistochemical expression of SOX9 (Sertoli cells), CYP11A1 (Leydig cells), and DDX4 (germ cells). Busulfan alone induced severe spermatogenic defects, including reduced sperm count and motility, increased abnormal morphology, marked histopathological damage, and decreased expression of SOX9, CYP11A1, and DDX4. In contrast, intravenous SVF treatment significantly improved sperm count, motility, and testicular architecture, and restored the expression of SOX9, CYP11A1, and DDX4 within two weeks. This is the first demonstration that intravenous SVF attenuates early busulfan-induced testicular injury, providing preliminary evidence for a rapid, culture-free approach as a less invasive alternative to local injection. However, longer follow-up and fertility studies are needed to confirm sustained spermatogenic restoration.

## Simple Summary

Chemotherapy can cause lasting damage to the testicles, leading to male infertility. In this study, we tested whether a cell-based therapy by using a mixture of cells obtained from fat tissue (called stromal vascular fraction, or SVF) could reduce testicular damage in rats treated with busulfan, a chemotherapy drug. We found that giving SVF intravenously immediately after busulfan exposure significantly improved sperm quality and testicular structure within two weeks. Our results suggest that this approach may offer a rapid and practical way to protect against chemotherapy-induced testicular injury. However, longer-term studies are needed to confirm whether these early improvements lead to lasting recovery of fertility.

## 1. Introduction

Damage to the testicles that affects sperm production is a major cause of male infertility. It can happen for several reasons, such as chemotherapy, toxins in the environment, infections, ischemia–reperfusion, and genetic defects [1,2,3]. Alkylating agents like busulfan have been widely used as a reliable way to cause severe germ cell depletion and testicular dysfunction in experiments [4]. Busulfan specifically targets cells in the seminiferous epithelium that are dividing quickly, which depletes spermatogonial stem cells and then causes Sertoli and Leydig cells to impair their function [5]. The pathological features that result from this treatment are hypergonadotropic hypogonadism (high follicle-stimulating hormone (FSH)/luteinizing hormone (LH) and low testosterone), azoospermia or severe oligozoospermia, increased germ cell apoptosis, oxidative stress, and chronic inflammation. These pathological features recapitulate key aspects of in many types of non-obstructive spermatogenic impairment [6,7]. Currently, very few treatment options exist for these conditions, and none have been proven to restore endogenous spermatogenesis [8].

Cell-based regenerative strategies have gotten a lot of attention in the last few years. Researchers have looked at mesenchymal stem cells (MSCs) from bone marrow, fat tissue, and umbilical cord in animal models of testicular injury [9,10,11]. For instance, giving MSCs through the veins or directly into the testicles has been shown to restore sperm production, improve hormone levels, and lower cell death in rodents that had been treated with busulfan [12]. The beneficial effects are mostly due to paracrine signaling, which includes the release of growth factors (like glial cell line-derived neurotrophic factor (GDNF), stem cell factor (SCF), and vascular endothelial growth factor (VEGF)), anti-inflammatory cytokines (like interleukin-10 (IL-10) and transforming growth factor-β (TGF-β)), and exosomes that change the local microenvironment and encourage the growth of stem cells in the body [11,13]. A recent review of the adipose-derived MSC secretome found that it helps seminiferous tubules grow back, lowers apoptosis, and speeds up the recovery of Leydig cells in testicular injury caused by chemicals [11]. However, there are a number of problems that MSC therapy will have to overcome to be used in people. To get enough MSCs, you often have to grow them outside of the body for several weeks, which delays treatment and raises costs [14,15]. Long-term culture can cause genetic instability and, in rare cases, tumorigenicity [14,16]. There are also ethical issues with using certain MSC sources, like fetal or embryonic tissues [17,18], that make them less useful, particularly due to concerns about consent and the moral implications of using these tissues in research and treatment. Researchers are looking for other cell types that are easy to get, safe, and work well because of these problems.

The stromal vascular fraction (SVF) is a group of different types of cells that come from breaking down adipose tissue with enzymes, so there is no need to grow them in culture [19]. SVF is made up of a mix of endothelial progenitor cells, pericytes, macrophages, fibroblasts, and adipose-derived stem cells (ADSCs) [20]. The ADSCs in SVF are very similar to cultured MSCs in many ways, such as their ability to be multipotent and affect the immune system [19]. However, SVF can be made in just 2–3 h from a small lipoaspirate, which makes it a “point-of-care” regenerative tool [21]. A recent in-depth review found that SVF has both immunomodulatory and proangiogenic effects [22]. It does so by modulating both innate and adaptive immune cells and releasing pro-angiogenic factors like VEGF, hepatocyte growth factor (HGF), and platelet-derived growth factor (PDGF) [22]. Researchers have looked into how SVF can help treat a wide range of diseases, such as osteoarthritis, heart attacks, peripheral neuropathy, and tissue fibrosis caused by radiation [23,24]. In most cases, the beneficial effects happen through paracrine mechanisms instead of direct differentiation. This leads to less inflammation, more blood vessel growth, and tissue regeneration [20,25].

Even though there is more and more evidence for these benefits, the use of SVF in testicular pathophysiology is still mostly unexplored. There have only been a few studies that looked at how uncultured SVF affects male reproductive organs. For example, Hekimoglu and Esrefoglu recently said that treating SVF inside the testicles helped with oxidative stress caused by busulfan in both testis tissue and serum [26]. It also raised the number of healthy sperm and lowered the number of abnormal tails in rats [26]. In a rat model of testicular torsion-detorsion, another study showed that autologous uncultured SVF protected the testis from ischemia–reperfusion injury and promoted spermatogenesis [27]. Of note, all of these previous SVF studies in testicular injury used local (intratesticular or intratissue) administration. However, to our knowledge, the efficacy of systemic (intravenous) delivery of SVF has not been evaluated in any testicular injury model, including busulfan-induced damage. Intravenous administration would be far less invasive, more easily repeatable, and more may offer greater clinical feasibility than direct testicular injection. More importantly, in studies using MSCs, intravenous injection has been proven to be an effective route for treating busulfan-induced testicular injury [12]. Given that busulfan-induced testicular damage shares key pathological features (oxidative stress, inflammation, germ cell apoptosis, Sertoli/Leydig cell dysfunction) with other etiologies, and based on the evidence that intravenous delivery is effective for other cell types, we hypothesized that intravenous infusion of SVF would similarly mitigate testicular injury in this model and could be broadly applicable to analogous spermatogenic disorders.

## 2. Materials and Methods

### 2.1. Isolation and Preparation of Stromal Vascular Fraction

Adipose tissue was obtained from the inguinal fat pads of healthy age-matched green fluorescent protein (GFP) transgenic Sprague-Dawley (SD) donor rats. SVF was isolated from several healthy age-matched GFP-transgenic SD donor rats. The isolated cells from all donors were pooled to minimize donor-to-donor variability, and all recipient animals received cells from the same pooled preparation. The excised fat was mechanically minced and placed in Hanks’ balanced salt solution (HBSS; GIBCO, Thermo Fisher Scientific, Carlsbad, CA, USA, C14175500BTI) containing 1% penicillin-streptomycin (GIBCO, Thermo Fisher Scientific, 15140-122), then washed repeatedly. The tissue was digested with 0.1% collagenase type I (Sigma-Aldrich, St. Louis, MO, USA, C1-28-100MG) in Dulbecco’s modified Eagle medium (DMEM; GE Hyclone, Logan, UT, USA, SH30021.01B) for 60 min at 37 °C under gentle shaking (200 rpm) in a constant-temperature shaking incubator. Enzyme activity was terminated by adding an equal volume of DMEM supplemented with 10% fetal bovine serum (FBS; GIBCO, Thermo Fisher Scientific, Carlsbad, CA, USA), which effectively neutralizes collagenase activity through dilution and serum-mediated inhibition. The digest was filtered through a 100 µm cell strainer to remove undigested debris and centrifuged at 400× *g* for 10 min. The supernatant was discarded, and the pelleted SVF was washed twice with phosphate-buffered saline (PBS), then resuspended in HBSS. The mean yield of SVF cells was 1.5 × 10^6^ cells/g of adipose tissue. Cell viability (>90%) was confirmed by trypan blue exclusion, for each preparation and cells were counted before use.

### 2.2. Characterization of SVF

To characterize the cellular composition of SVF, freshly isolated cells were analyzed by flow cytometry. Briefly, 5 × 10^5^ cells were suspended in flow cytometry tubes. Unstained samples served as negative controls. Antibodies against CD90 phycoerythrin-cyanine7 (PE-Cy7; Elabscience, Houston, TX, USA), CD31 allophycocyanin (APC; R&D, Minneapolis, MN, USA), and CD45 APC-Cy7 (Elabscience, Houston, TX, USA) were added to a final volume of 100 μL. The samples were incubated in the dark at 4 °C for 2 h, then 1 mL of PBS was added, and the cells were centrifuged at 300× *g* for 5 min. The supernatant was discarded, and the pellet was resuspended in 200 μL PBS for acquisition. Data were collected on a flow cytometer (Aisen Biology (Hangzhou) Co., Ltd., D2060R, Hangzhou, China) and analyzed using FlowJo v10 (BD Biosciences, Ashland, OR, USA).

Flow cytometry data were analyzed using a sequential gating strategy. Initially, cellular debris and noncellular events were excluded based on forward scatter-area (FSC-A) and side scatter-area (SSC-A) parameters to isolate intact cells. Next, viable cells were identified by excluding dead cells using a fixable live/dead staining dye (Ghost Dye™ Blue 516, BioLegend, San Diego, CA, USA, Cat. No. 425303), which ensures accurate discrimination and removal of non-viable events. From this viable population, doublets and cell clusters were further removed from the analysis using FSC-A versus FSC-height (FSC-H) plots. The viable single-cell population was then subdivided into hematopoietic (CD45^+^) and non-hematopoietic (CD45^−^) populations based on CD45 expression. Subsequently, the CD45^−^ non-hematopoietic fraction was analyzed for the co-expression of CD31 (endothelial cell marker) and CD90 (mesenchymal stem cell marker) using a bivariate quadrant plot. This gating strategy clearly separates distinct subpopulations, and the representative flow cytometry plots illustrating this sequential strategy are presented in Supplementary Figure S1.

To further dissect the cellular heterogeneity of SVF, we performed single-cell RNA sequencing on three independent preparations (biological replicates, *n* = 3). One of these preparations was previously analyzed in our recent study [20]; the other two were generated specifically for this work to assess batch-to-batch reproducibility. Sequencing was performed using the 10× Genomics platform. Demultiplexing and alignment of raw sequencing reads were performed using CellRanger (version 8.0.0). After quality control, data normalization, and clustering analysis were carried out using the Seurat pipeline (version 5.1.0) in R (version 4.3.2), distinct cell subpopulations were identified based on t-distributed stochastic neighbor embedding (t-SNE) dimensionality reduction and annotated according to canonical marker genes. Doublets were detected and excluded using DoubletFinder (version 2.0.4). For quality control, cells with <200 detected genes or >20% mitochondrial reads were excluded from downstream analysis. Cells were clustered and marker genes for each cell type were used to annotate the cells. A full list of marker genes used for annotation is provided in Table 1.

### 2.3. Animals and Experimental Design

Adult male SD rats (8 weeks old, 240–260 g) were purchased from Guangdong Weitong Lihua Laboratory Animal Technology Company (Guangdong, China). Animals were given a one-week acclimatization period before the start of experiments. Rats were housed three per cage under standard conditions (12 h light/dark cycle, temperature 23 ± 3 °C, relative humidity 30–70%, food and water ad libitum). All procedures were conducted in accordance with the regulations of the Animal Ethics Committee (protocol no. 2022d152) of the South China Agricultural University Laboratory.

Rats were randomly divided into three groups (*n* = 6 per group, except where indicated otherwise in figure legends). Sample sizes were chosen based on previous studies in the field, which commonly used *n* = 6–8 per group for sperm analysis [28] and *n* = 3–6 for histological evaluations [29]; *n* = 3 was selected for detailed morphological analysis to minimize animal use [29]. Sample sizes varied across different analyses due to the following reasons: (a) *n* = 3 for histopathological and immunohistochemical analyses, which is standard for detailed morphological evaluation; For histopathological quantification (JMTBS, STD, GET), all 6 animals in the Busulfan and SVF-V groups were analyzed, whereas 3 representative animals from the Control group were randomly selected in a blinded manner. For immunohistochemical staining, 3 animals per group were used for detailed morphological evaluation. (b) *n* = 5–6 for sperm analysis and hormone measurements, where larger sample numbers were available; (c) *n* = 4 for some hormone assays due to insufficient serum volume in certain animals. The Control group (Control) received vehicle only. The busulfan group (Busulfan) received a single intraperitoneal injection of busulfan (15 mg/kg; Yuanye Bio-Technology, Shanghai, China) dissolved in dimethyl sulfoxide (DMSO)/corn oil (1:9), as described in previous studies [30,31]. The Busulfan + SVF group [(SVF-V)] received busulfan as above, followed immediately by intravenous injection of 2 × 10^6^ SVF cells suspended in 200 µL PBS via the tail vein. The Normal control and Busulfan groups received an equivalent volume of vehicle (200 µL PBS) via the tail vein to control for the injection procedure. All animals were euthanized two weeks after busulfan treatment. Blood was collected by cardiac puncture, and testes and epididymides were collected for further studies.

### 2.4. Hormone Assays

Blood was collected from the inferior vena cava and then centrifuged at 2000× *g* for 15 min at 4 °C. The serum was subsequently stored at −80 °C until analysis. Serum levels of FSH, LH, testosterone, and estradiol were measured using commercially available enzyme-linked immunosorbent assay (ELISA) kits (rat FSH/LH ELISA kit, MyBioSource, San Diego, CA, USA; testosterone and estradiol ELISA kits, Cayman Chemical, Ann Arbor, MI, USA) according to the manufacturers’ instructions. Estradiol was included because it is produced in the testis through aromatization of testosterone and plays a role in spermatogenesis and Leydig cell function [32].

### 2.5. Sperm Analysis

Spermatozoa were collected from the right cauda epididymis by incising the tissue in 1 mL of pre-warmed PBS and allowing sperm to swim out for 15 min. Sperm density was determined using a hemocytometer. Motility (progressive and total motility) was assessed manually under a light microscope (400×) [33]. For morphology, smears were stained with Diff-Quick and at least 200 sperm per animal were evaluated for abnormalities using a 500-count board.

### 2.6. Epididymal Sperm Content and Histology

The left epididymis was fixed and processed for paraffin sectioning. Hematoxylin and eosin (H&E) staining was performed to visualize sperm presence in the epididymal lumen.

### 2.7. Testicular Histopathology

Testes were fixed in Bouin’s solution, embedded in paraffin, sectioned at 4 µm, and stained with H&E. For each animal, 10 cross-sections of seminiferous tubules were randomly selected. Three parameters were assessed: the Johnsen mean tubular biopsy score (JMTBS), a 10-point scoring system (1 = no germ cells, 10 = complete spermatogenesis); the seminiferous tubule diameter (STD) measured using Case Viewer software (version 2.4.0.119028) was developed by 3DHISTECH Ltd. (Budapest, Hungary); and the germinal epithelial thickness (GET) measured from the basement membrane to the lumen. Animal (not individual tubules or microscopic fields) was used as the statistical unit for all analyses. For tubule-level measurements (JMTBS, STD, GET), we first calculated the mean value per animal from multiple tubules, and these animal-level means were then used for statistical comparisons.

### 2.8. Immunohistochemistry

Paraffin sections (4 µm) were deparaffinized in xylene and rehydrated through graded ethanol. Antigen retrieval was performed by heating the sections in citrate buffer (pH 6.0) for 15 min in a microwave oven. Endogenous peroxidase activity was blocked with 3% hydrogen peroxide in methanol for 10 min. Non-specific binding was blocked with 5% normal goat serum for 30 min at room temperature. Sections were then incubated overnight at 4 °C with the following primary antibodies: rabbit anti-SOX9 (1:200, catalog No. ab185966, Abcam, Cambridge, UK), rabbit anti-CYP11A1 (1:100, catalogue No. sc-18044 Santa Cruz, Inc., Dallas, TX, USA), and mouse anti-DDX4 (1:250, catalogue No. 17545-1-AP, Proteintech, Wuhan, China). Antibody specificity was validated by the manufacturers and confirmed by positive controls (normal rat testis) and negative controls (replacement of primary antibody with normal serum). After washing three times with PBS, sections were incubated with biotinylated secondary antibody (goat anti-rabbit or goat anti-mouse IgG, 1:200, Vector Laboratories, Inc., Newark, CA, USA) for 30 min at 37 °C. Subsequently, the sections were treated with avidin-biotin-peroxidase complex (ABC; ABC kit, Vector Laboratories) for 30 min at 37 °C. The reaction was visualized using 3,3′-diaminobenzidine (DAB) as the chromogen, resulting in a brown precipitate. Sections were counterstained with hematoxylin, dehydrated, cleared, and mounted. For each staining run, negative controls were included in which the primary antibody was replaced with normal serum. Images were captured using a light microscope (Olympus, Corporation, Tokyo, Japan).

Quantitative analysis was performed using GraphPad Prism 8.0.1. The percentage of SOX9-positive tubules was calculated from at least 25 tubules per section. The integrated optical density of CYP11A1 was measured in five randomly selected high-power fields per section, and DDX4-positive germ cells were counted in 25 tubules per animal with the mean per animal used as the statistical unit. All quantitative analyses were performed by an investigator blinded to the experimental groups.

### 2.9. GFP Detection

To assess whether intravenously infused SVF cells engrafted into the testicular interstitium, testicular, liver, and lung tissues were harvested from SVF-treated rats at two weeks post-injection, embedded in optimal cutting temperature (OCT) compound, and snap-frozen in liquid nitrogen. Frozen sections (10 μm) were prepared using a cryostat and mounted onto glass slides. GFP fluorescence was visualized directly under a fluorescence microscope (Carl Zeiss Microscopy GmbH, Jena, Germany) without additional staining. Testicular sections from normal (non-GFP) SD rats served as negative controls. As a positive control, GFP-positive SVF cells cultured in vitro for 6 days were used to confirm the detection of the GFP signal.

### 2.10. Transmission Electron Microscopy

A small piece (1 mm^3^) of fresh testicular tissue was taken from each animal and fixed in 2.5% glutaraldehyde at 4 °C in the dark. The tissue was then transferred to 1% osmium tetroxide for post-fixation at room temperature in the dark for 2 h. After fixation, the samples were dehydrated at room temperature and embedded in epoxy resin. Ultrathin sections (70 nm) were stained with uranyl acetate and lead citrate, then examined under a transmission electron microscope (JEOL Ltd., Akishima, Tokyo, Japan). Images were captured at indicated magnifications.

### 2.11. Statistical Analysis

All statistical analyses were performed using GraphPad Prism 8.0.1 software. Data are presented as mean ± standard deviation (SD). For comparisons among three groups, one-way analysis of variance (ANOVA) followed by Tukey’s post hoc test was applied. When equal variances were not assumed, Brown–Forsythe and Welch ANOVA was performed. Non-normally distributed data were analyzed using the Kruskal–Wallis test. A *p*-value < 0.05 was considered statistically significant. Detailed sample sizes (*n*) are indicated in each figure legend.

## 3. Results

### 3.1. Cellular Composition and Heterogeneity of the Isolated SVF Were Identified

**Figure 1:**
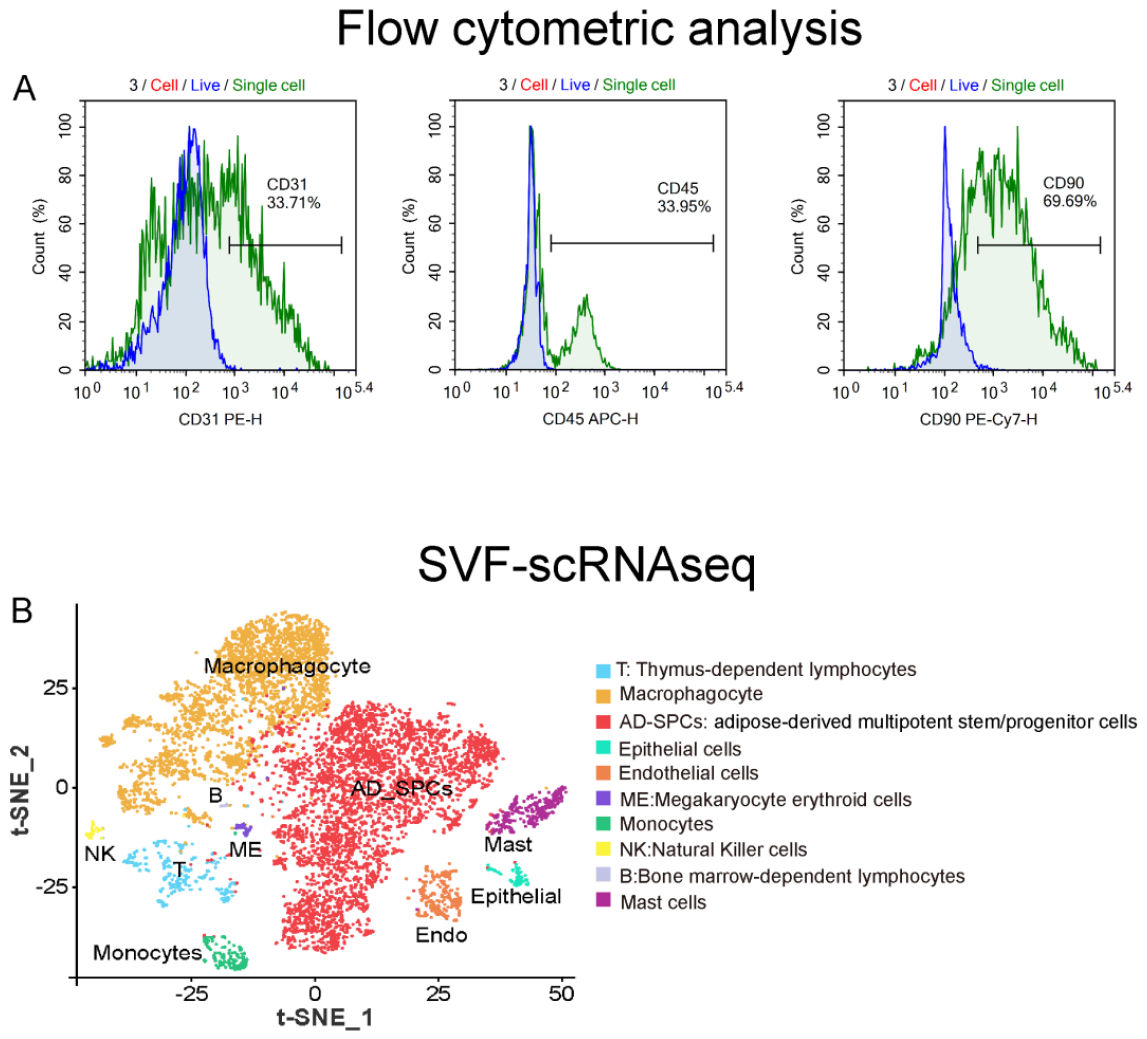
Cellular composition and heterogeneity of the isolated SVF. (**A**) Flow cytometry analysis of surface marker expression for CD90, CD31, and CD45 (Green histograms). Isotype-matched controls (Blue histograms) were used to define the positive gate. Numbers indicate the percentage of cells positive for each marker. (**B**) t-SNE visualization of single-cell RNA-seq data showing 10 distinct cell subsets. Each dot represents a single cell, and different colors indicate cell subpopulations.

To determine the cellular composition of the isolated SVF, surface marker expression was analyzed using flow cytometry. SVF cells were stained with antibodies to CD31 (endothelial cell marker), CD45 (hematopoietic lineage marker) and CD90 (MSC marker). The results showed heterogeneity in marker expression within the SVF population. In particular, 33.71% of SVF cells were CD31+ (endothelial cell marker, Figure 1A), 33.95% were CD45+ (hematopoietic cell marker, Figure 1A), and 69.69% were CD90+ (MSC marker, Figure 1A). These results show that the isolated SVF is a mix of endothelial, hematopoietic and mesenchymal stromal cell populations.

To further dissect the cellular heterogeneity of SVF, we performed single-cell RNA sequencing on three independent preparations. Figure 1B shows a representative t-SNE plot from one preparation, revealing 10 distinct cell subsets. To assess reproducibility across batches, t-SNE plots from the remaining two preparations are presented in Supplementary Figure S2A,B, and the proportions of each subset across the remaining two batches are summarized in Supplementary Table S1. The same 10 major cell populations were consistently identified across all three batches, indicating that the SVF cellular composition is largely consistent across biological replicates.

### 3.2. SVF Increases Testosterone Without Affecting Other Serum Hormone Levels

**Figure 2:**
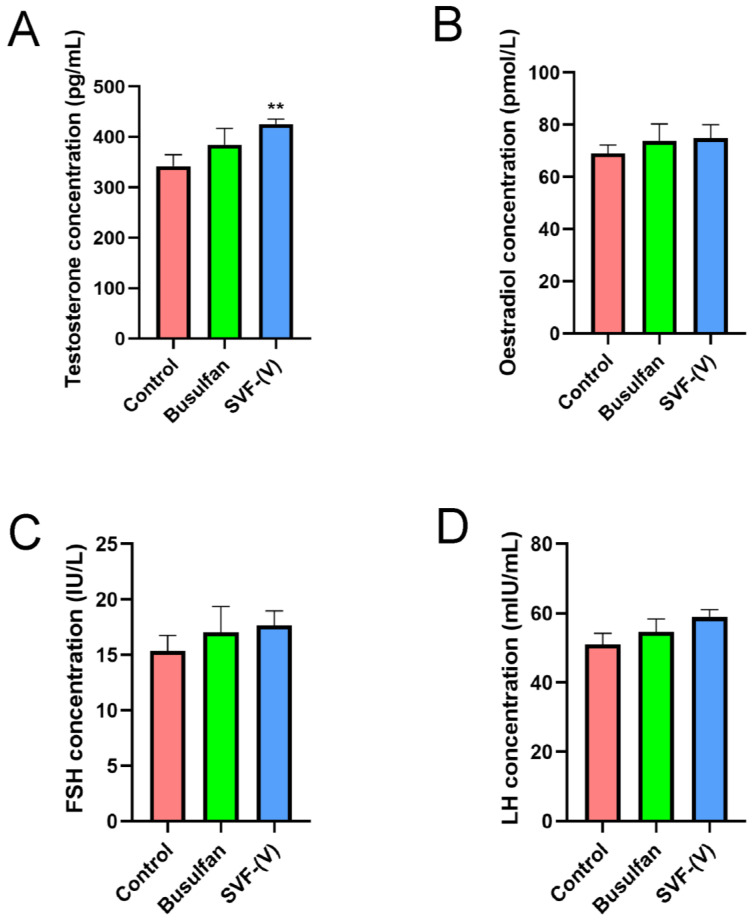
Effects of SVF on serum hormone levels. (**A**) Testosterone concentration: ** *p* < 0.01 vs. Busulfan group. (**B**) Estradiol, (**C**) FSH, (**D**) LH. No significant differences were observed among groups for estradiol, FSH, LH, or for testosterone between Control and Busulfan groups. Data are mean ± SD. For (**A**–**C**), *n* = 5 per group; for (**D**), *n* = 4 per group.

Serum hormone levels were measured to assess the endocrine status (Figure 2). At the two-week time point, busulfan alone did not significantly alter serum FSH, LH, estradiol, or testosterone concentrations compared with the Control group (Figure 2A–D). However, SVF treatment significantly increased testosterone concentration compared with the Busulfan group (*p* < 0.01; Figure 2A). Estradiol, FSH, and LH levels remained unchanged after SVF administration, showing no significant differences among the three groups (Figure 2B–D). These data indicate that at two weeks post-injury, the only hormonal change induced by SVF is a selective increase in testosterone, independent of alterations in gonadotropins or estradiol.

### 3.3. SVF Ameliorates Histopathological Changes in Busulfan-Damaged Testes

**Figure 3:**
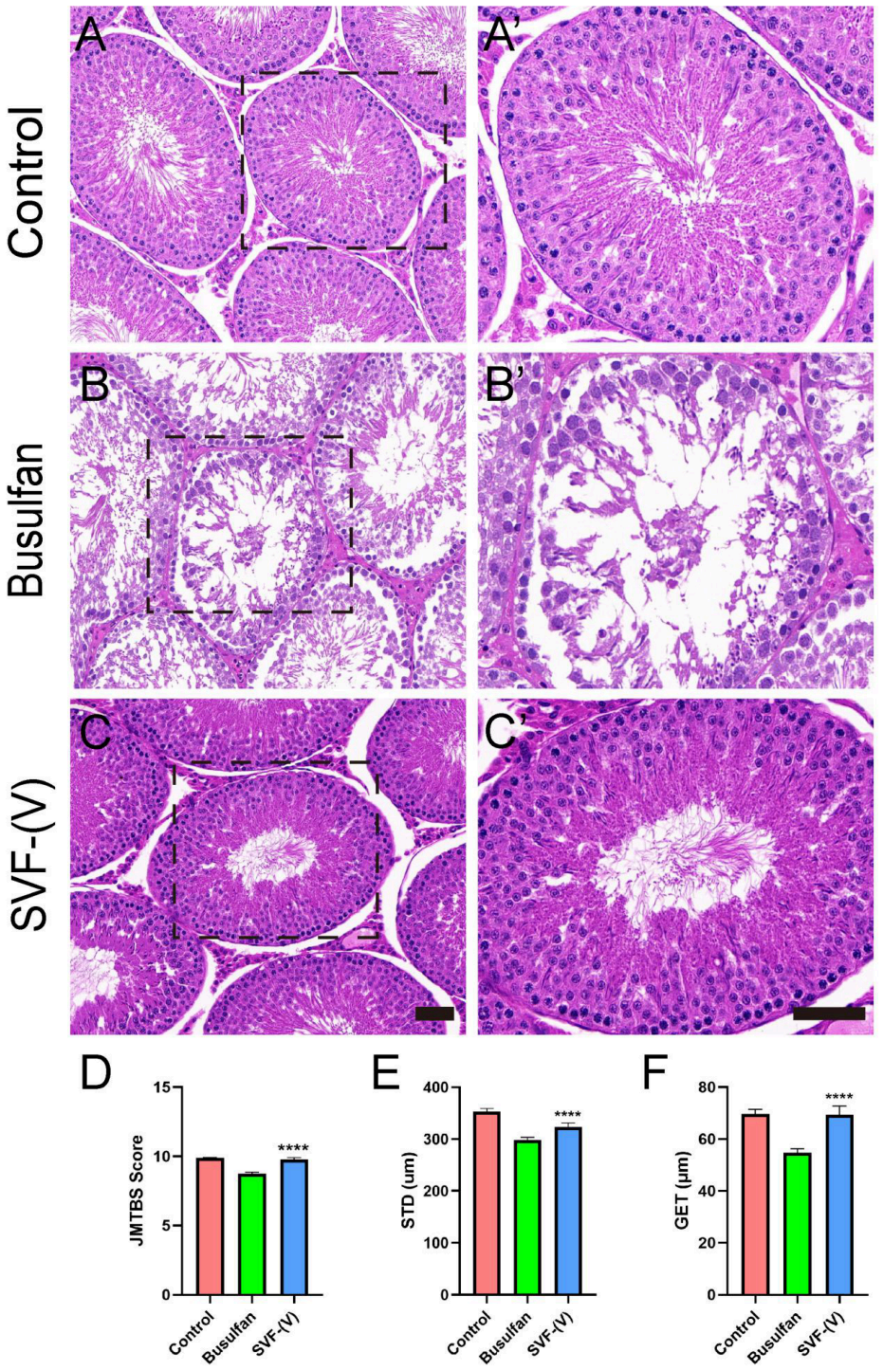
SVF promotes testicular injury repair. (**A**–**C**) Representative H&E staining of testicular sections. (**A’**,**B’**,**C’**) High-magnification views of the insets (indicated by dashed boxes) in (**A**), (**B**), and (**C**), respectively (scale bars = 50 μm). (**D**) JMTBS, (**E**) STD, and (**F**) GET: **** *p* < 0.0001 vs. Busulfan group for all parameters. Data are mean ± SD. Control group, *n* = 3; Busulfan and SVF-V groups, *n* = 6 each.

Testicular sections stained with H&E were evaluated using three quantitative parameters (Figure 3). The Busulfan group exhibited severe seminiferous tubule atrophy, germ cell loss, and disrupted architecture (Figure 3B,B’). In contrast, SVF-treated rats showed a remarkable recovery, with many tubules containing organized layers of germ cells and spermatids (Figure 3C,C’), resembling the Control group (Figure 3A,A’). The JMTBS was dramatically reduced by busulfan and significantly increased by SVF (*p* < 0.0001 vs. Busulfan; Figure 3D). Similarly, both STD and GET were ameliorated after SVF injection (*p* < 0.0001 vs. Busulfan for both; Figure 3E,F). These results demonstrate that SVF effectively ameliorates busulfan-induced testicular histopathology within two weeks.

### 3.4. SVF Improves Epididymal Sperm Content

**Figure 4:**
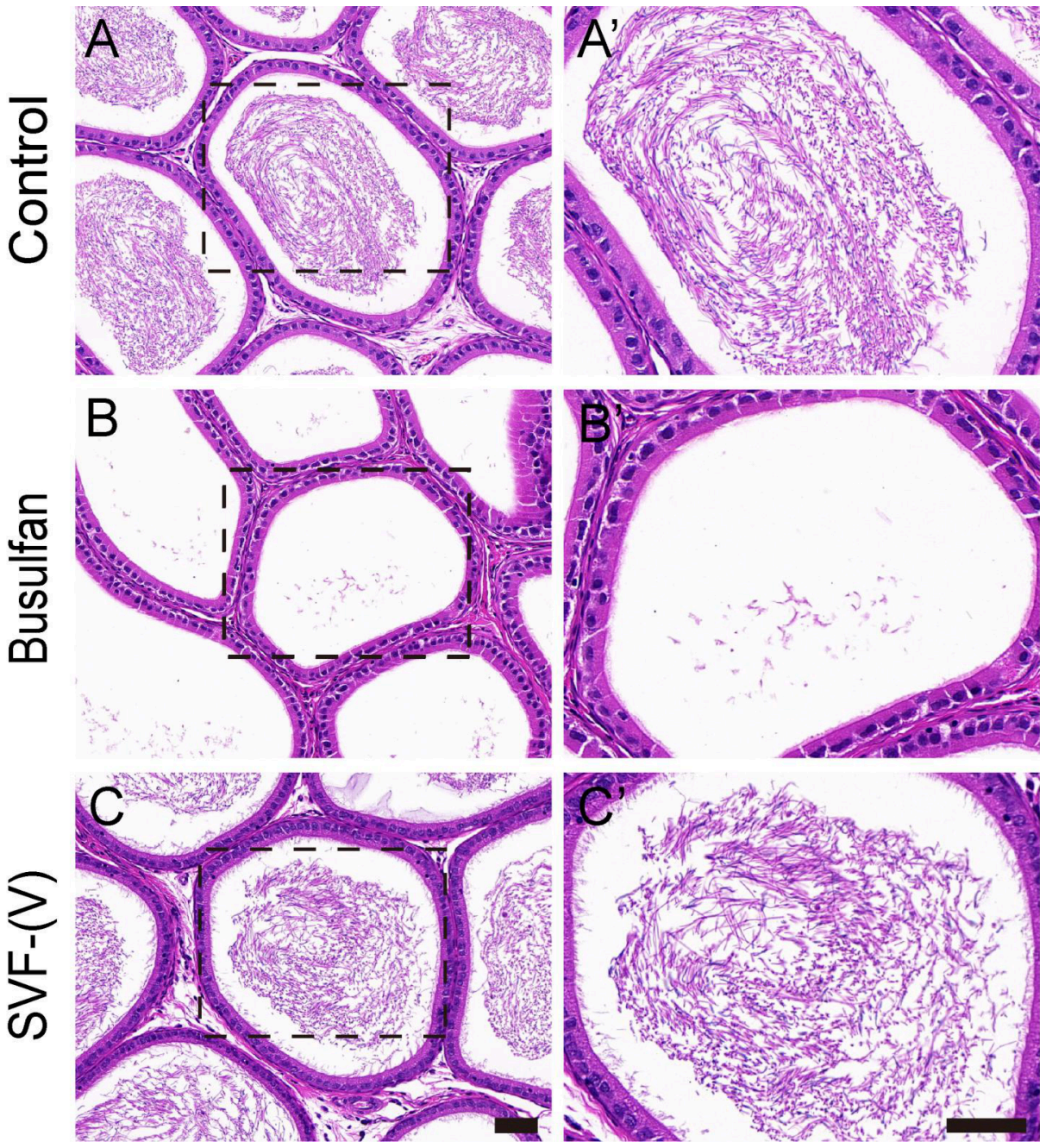
Restoration of epididymal sperm content by SVF. (**A**–**C**) Representative H&E staining of sections of epididymis. (**A’**,**B’**,**C’**) High-magnification views of the insets (indicated by dashed boxes) in (**A**), (**B**), and (**C**), respectively. *n* = 6 per group. Scale bars = 50 μm.

Epididymal histology is shown in Figure 4. The busulfan-treated epididymis contained few or no sperm in the lumen (Figure 4B,B’), whereas the SVF group exhibited abundant sperm clusters (Figure 4C,C’), similar to the normal control (Figure 4A,A’). This qualitative observation was supported by the sperm count data presented below.

### 3.5. SVF Improves Sperm Quantity and Quality

**Figure 5:**
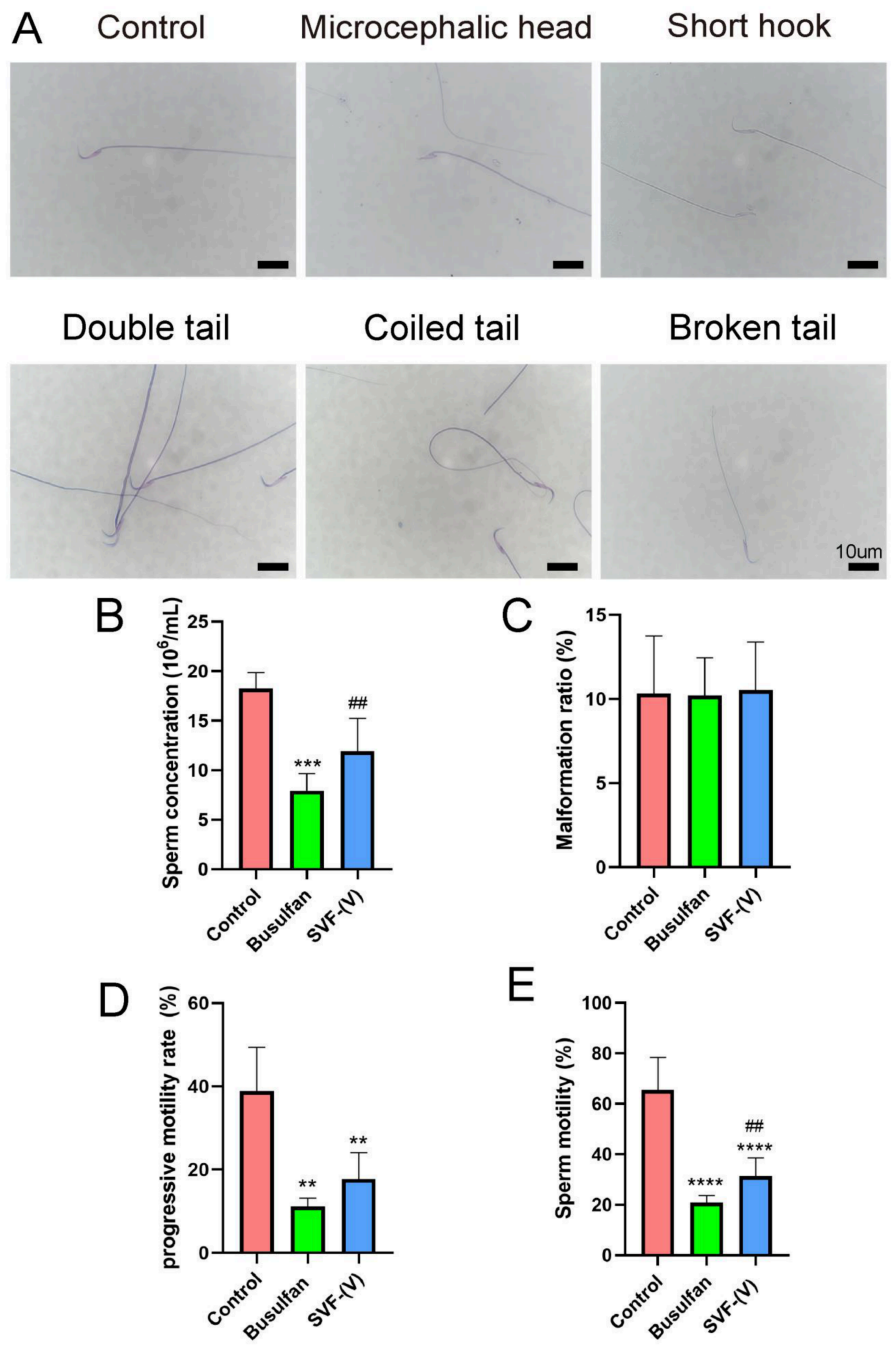
SVF improves sperm quantity and quality. (**A**) Control and common abnormal morphology of rat sperm (scale bar = 10 μm). (**B**) Sperm density: *** *p* < 0.001 vs. Control group; ## *p* < 0.01 vs. Busulfan group. (**C**) Sperm morphological abnormality rate, determined using a 500-count board. (**D**) Progressive motility rate: ** *p* < 0.01 vs. Control group. (**E**) Total sperm motility: **** *p* < 0.0001 vs. Control group; ## *p* < 0.01 vs. Busulfan group. Data are mean ± SD (*n* = 6 per group).

Sperm parameters were analyzed from the cauda epididymis (Figure 5). Representative images of normal and abnormal sperm morphology are shown (Figure 5A). Busulfan caused a drastic decrease in sperm density, which was significantly increased by SVF treatment (*** *p* < 0.001 vs. Control; ## *p* < 0.01 vs. Busulfan; Figure 5B). The sperm malformation rate was elevated after busulfan, and SVF treatment showed a trend toward reduction; however, this difference did not reach statistical significance (Figure 5C). Both progressive motility (Figure 5D) and total sperm motility (Figure 5E) were impaired by busulfan; SVF treatment significantly improved total motility (**** *p* < 0.0001 vs. Control; ## *p* < 0.01 vs. Busulfan). These findings confirm that SVF improves not only the number but also the functional quality of sperm as early as two weeks after treatment.

### 3.6. SVF Upregulates the Expression of Key Testicular Cell Markers as Shown by Immunohistochemistry

**Figure 6:**
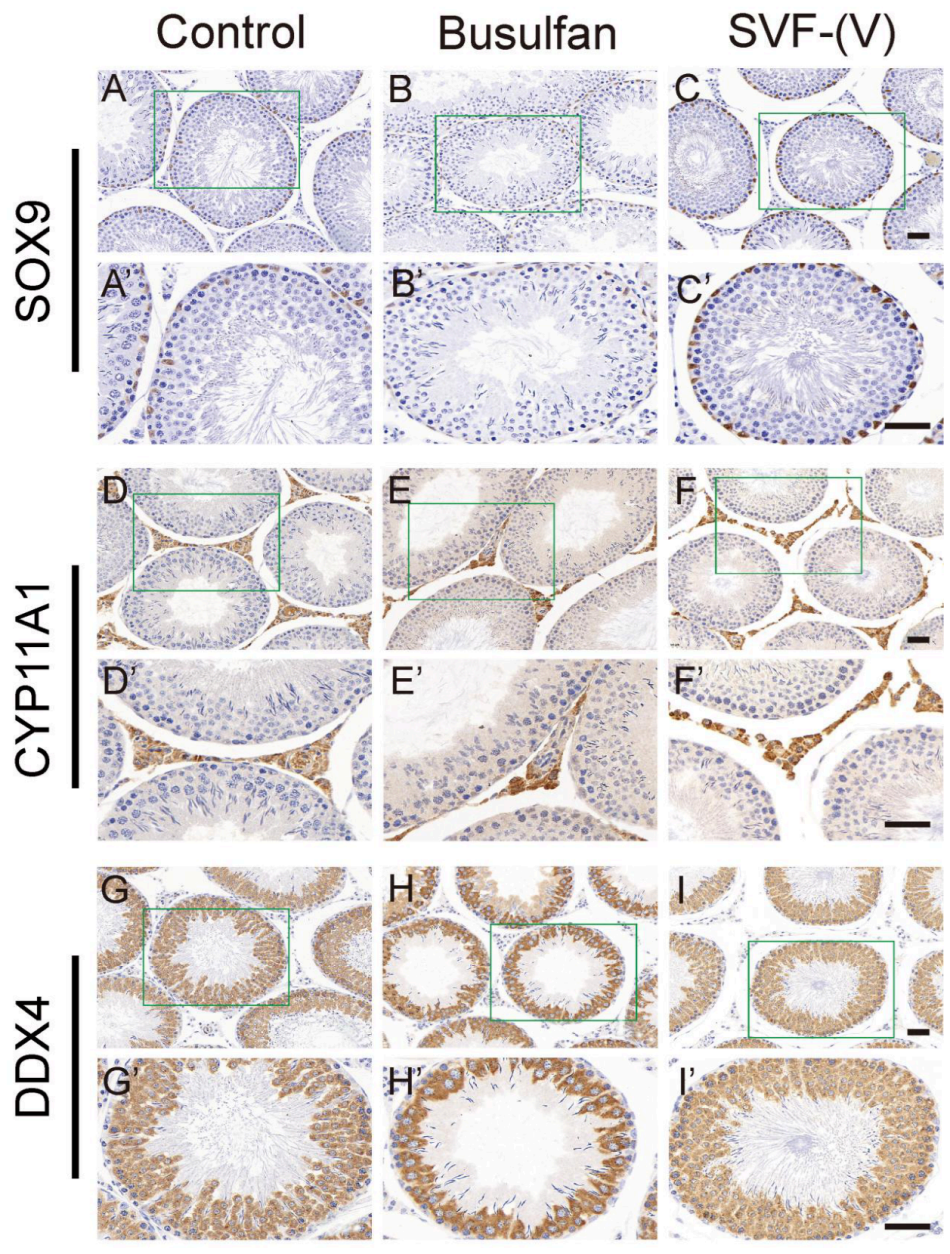

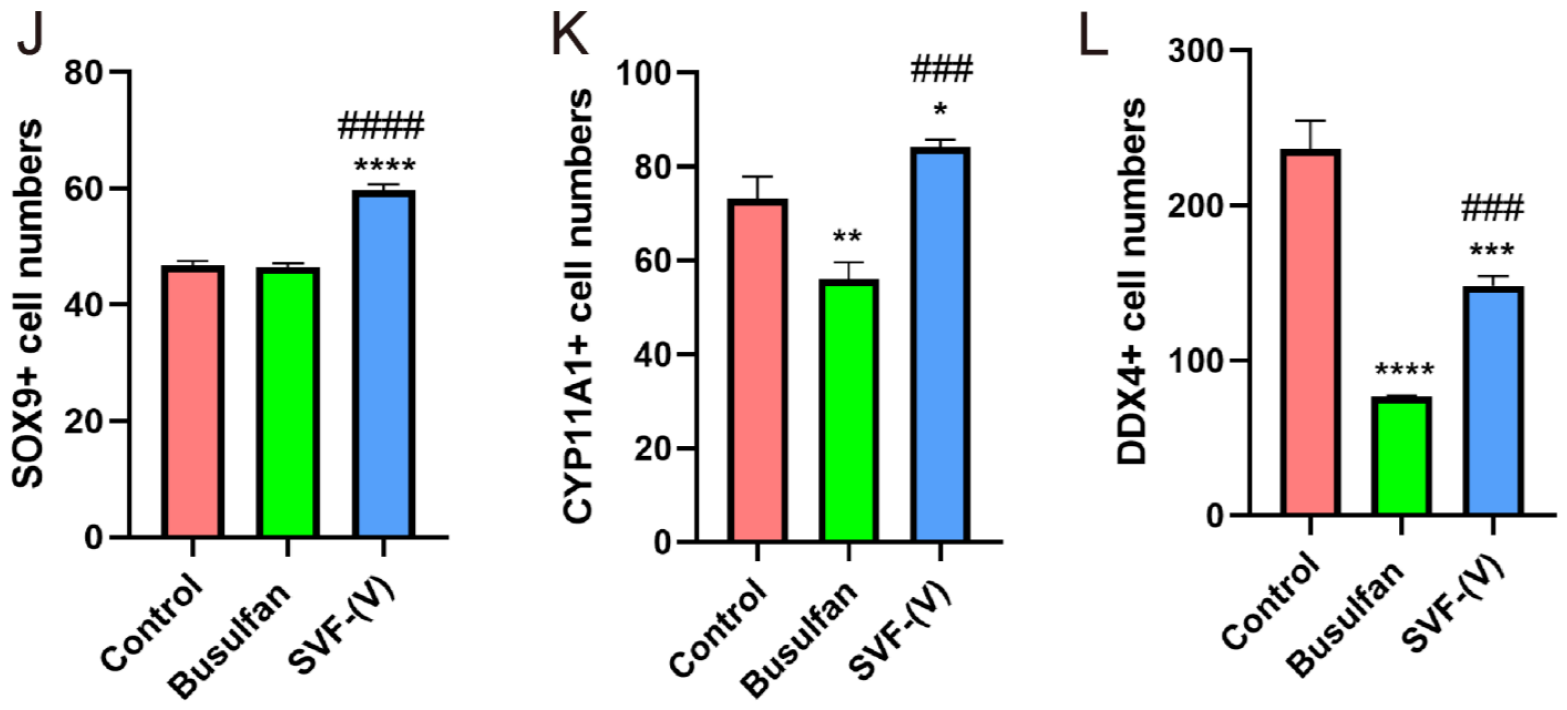
Immunohistochemical expression of SOX9, CYP11A1, and DDX4 in the SVF-treated group. (**A**–**C**) Morphology of SOX9^+^ cells; (**A’**–**C’**) high-magnification views of the corresponding insets (indicated by green boxes). (**D**–**F**) Morphology of CYP11A1^+^ cells; (**D’**–**F’**) high-magnification views of the corresponding insets (indicated by green boxes). (**G**–**I**) Morphology of DDX4^+^ cells; (**G’**–**I’**) high-magnification views of the corresponding insets (indicated by green boxes). (**J**) Quantitative analysis of SOX9^+^ cells: **** *p* < 0.0001 vs. Control group; #### *p* < 0.0001 vs. Busulfan group. (**K**) Quantitative analysis of CYP11A1^+^ cells: * *p* < 0.05, ** *p* < 0.01 vs. Control group; ### *p* < 0.001 vs. Busulfan group. (**L**) Quantitative analysis of DDX4^+^ cells: *** *p* < 0.001 and **** *p* < 0.0001 vs. Control group; ### *p* < 0.001 vs. Busulfan group. Data are mean ± SD; *n* = 3 per group. Scale bars = 50 μm.

Immunohistochemical staining was performed for SOX9 (Sertoli cells), CYP11A1 (Leydig cells), and DDX4 (germ cells) using the ABC-DAB method (Figure 6). In the Busulfan group, SOX9-positive Sertoli cells were markedly reduced in number and staining intensity (Figure 6B), and quantitative analysis showed a significant decrease in the percentage of SOX9-positive tubules compared with the Control group (*p* < 0.0001). SVF treatment restored SOX9 expression to levels significantly higher than those in the Busulfan group (#### *p* < 0.0001; Figure 6J). CYP11A1 expression in Leydig cells followed a similar pattern: busulfan reduced the integrated optical density (IOD) (* *p* < 0.05, ** *p* < 0.01 vs. Control), and SVF treatment significantly increased it (### *p* < 0.001 vs. Busulfan; Figure 6K). DDX4-positive germ cells were almost absent after busulfan injury (*** *p* < 0.001, **** *p* < 0.0001 vs. Control), and SVF administration significantly restored the number of DDX4-positive cells per tubule (### *p* < 0.001 vs. Busulfan; Figure 6L). These results indicate that SVF supports the survival and/or differentiation of Sertoli, Leydig, and germ cells within a two-week window.

### 3.7. Ultrastructural Analysis Reveals That SVF Attenuates Busulfan-Induced Subcellular Damage

**Figure 7:**
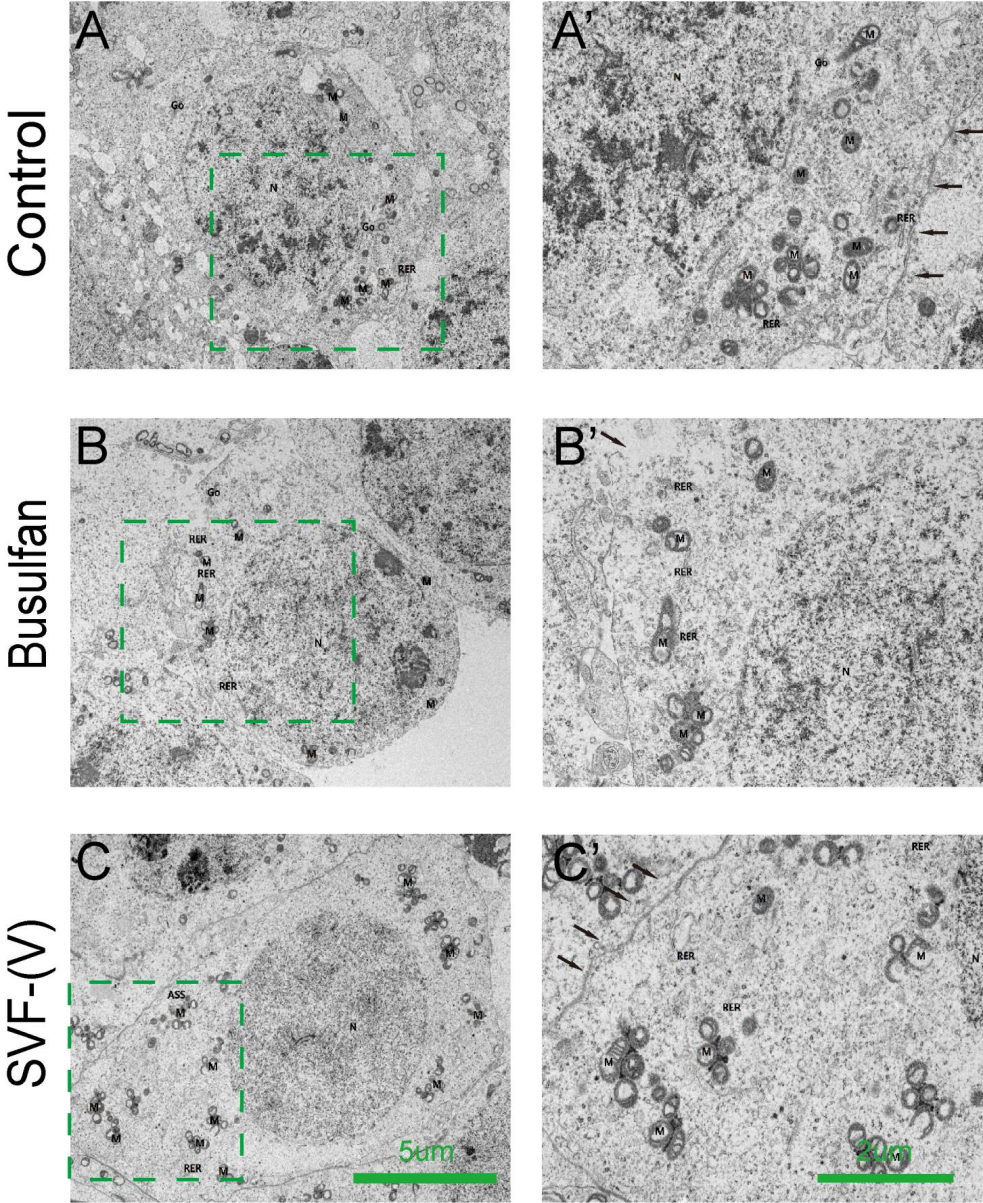
TEM images of primary spermatocytes. (**A**,**A’**) Control group. (**B**,**B’**) Busulfan group. (**C**,**C’**) SVF-V group. (**A’**,**B’**,**C’**) High-magnification views of the insets (indicated by dashed boxes) in (**A**), (**B**), and (**C**), respectively. Mitochondria (M) are indicated. Scale bars in (**A**–**C**) = 5 μm; scale bars in (**A’**–**C’**) = 2 µm.

Transmission electron microscopy (TEM) was performed to evaluate ultrastructural changes in primary spermatocytes. In the Control group (Figure 7A,A’), cell membranes were intact and continuous (arrows in Figure 7A’), the cytoplasm was uniform with a normal number of organelles, and abundant mitochondria (indicated by “M” in Figure 7) were mostly uniform in size, with intact membranes, homogeneous matrix, and tubular-vesicular cristae. In the Busulfan group (Figure 7B,B’), spermatocytes displayed pronounced structural damage: cell membranes were extensively discontinuous (arrow in Figure 7B’), the cytoplasm appeared sparse with reduced organelles, and mitochondria were markedly reduced in number, with some markedly dilated. In the SVF-V group (Figure 7C,C’), spermatocytes showed relatively preserved ultrastructure: cell membranes were intact and continuous (arrows in Figure 7C’), the cytoplasm was uniform with a normal number of evenly distributed organelles, and mitochondria were mostly normal, uniform in size, with intact membranes, homogeneous matrix, and tubular-vesicular cristae. These ultrastructural data corroborate the light microscopy findings and demonstrate that SVF protects testicular cells from busulfan-induced degeneration as early as two weeks post-treatment.

### 3.8. GFP Tracking of Intravenously Injected SVF Cells

To assess the biodistribution of intravenously infused SVF cells, we examined GFP fluorescence in testicular, lung, and liver tissues from SVF-treated rats at two weeks post-injection (Supplementary Figure S3). Abundant GFP-positive cells were detected in the lung, confirming successful cell delivery. Sparse GFP-positive cells were observed in the liver. However, no GFP-positive cells were detected in testicular sections from SVF-treated animals, comparable to the negative control (normal testis). These findings indicate that intravenously administered SVF cells did not engraft in the testis within the two-week observation period.

## 4. Discussion

This study showed that giving rats a single intravenous injection of uncultured SVF right after they were exposed to busulfan could ameliorate testicular damage in two weeks. Our method of systemic intravenous delivery is less invasive and may offer advantages for potential clinical application over the previously reported intratesticular route [26]. SVF treatment improved sperm count and quality, improved histopathological scores, and increased the expression of Sertoli (SOX9), Leydig (CYP11A1), and germ cell (DDX4) markers. These results suggest that SVF, a point-of-care cell preparation, may rapidly attenuate busulfan-induced spermatogenic impairment. Busulfan-induced testicular damage reflects various common pathological mechanisms, including oxidative stress, inflammation, germ cell apoptosis, and supporting cell dysfunction. Consequently, our findings may have broader implications for addressing spermatogenic impairment of diverse etiologies [1,2,3,27].

The busulfan-induced rat model utilized in this study emulates critical features of diverse forms of non-obstructive spermatogenic impairment, including the depletion of spermatogonial stem cells and disruption of the seminiferous epithelium [26,27]. Our initial data corroborate previous findings [26,27]. The two-week duration is substantially shorter than standard regeneration studies, and is insufficient to capture complete spermatogenic regeneration, as a full spermatogenic cycle in rats requires approximately 52 days. [12]. Nonetheless, we recorded significant recovery. This rapid effect suggests that SVF may primarily operates through short-term paracrine and immunomodulatory mechanisms, rather than by directly differentiating cells or gradually supporting stem cells. SVF is a diverse group of cells that includes ADSCs, endothelial cells, pericytes, and immune cells [20]. SVF cells may release many growth factors and cytokines, such as SCF and VEGF [34]. SCF promotes communication between Sertoli cells and germ cells, which is necessary for normal spermatogenesis [35]. In a testicular torsion-detorsion model, SVF injection increased the testicular expression of SCF, leading to reduced injury and enhanced spermatogenesis [27]. In the present study, SVF may also increase SCF levels in testicular cells, which supports the idea that it could work as a paracrine agent. VEGF, on the other hand, helps the testicles grow new blood vessels, which provides the healing tubules more oxygen and nutrients [36]. These factors may work together to create a pro-regenerative microenvironment that may support germ cell survival, proliferation, and differentiation into different types of cells.

Immunomodulation may play a significant role [37]. Busulfan induces a sterile inflammatory response in the testis, characterized by macrophage infiltration and the presence of pro-inflammatory cytokines (tumor necrosis factor-α (TNF-α) and IL-1β) [7]. SVF contains regulatory T cells and M2-like macrophages that may change the microenvironment to one that is anti-inflammatory and pro-repair [20,37]; this prevents additional harm to the seminiferous epithelium.

SVF population used in this study was highly heterogeneous, with macrophages (36.8%) and adipose stem/progenitor cells (47.59%) being the majority of the cell population. Adipose stem/progenitor cells are a rich source of paracrine factors and may release many growth factors and cytokines such as SCF and VEGF [34]. SCF promotes communication between Sertoli cells and germ cells, which is necessary for normal spermatogenesis [35]. In a testicular torsion-detorsion model, SVF injection increased the testicular expression of SCF, which resulted in reduced injury and improved spermatogenesis [27]. In the present study, SVF may also increase the levels of SCF in testicular cells, supporting the idea that it could work as a paracrine agent.; meanwhile, VEGF induces angiogenesis in the testis, which may favor the healing tubules with more oxygen and nutrients [36]. Moreover, macrophages, especially M2-like macrophages, are known to have anti-inflammatory and pro-repair effects. The observed regenerative effects may be due to the combined action of multiple cell types. Whether specific subsets are mainly responsible for the therapeutic effects, or whether the observed benefit requires the synergistic action of multiple populations, remains to be determined.

It is important to note that the proposed paracrine and immunomodulatory mechanisms are hypotheses based on the observed outcomes and the known biology of SVF, rather than direct evidence. We did not measure cytokines, growth factors, oxidative stress markers, inflammatory mediators, or signaling pathways in this study. Therefore, these mechanistic interpretations should be considered speculative and require direct validation in future studies.

There is still a chance that a small number of SVF cells could be directly integrated into the testicular interstitium. However, to assess whether intravenously infused SVF cells directly integrated into the testicular interstitium, we tracked donor SVF cells derived from GFP transgenic SD rats. At two weeks post injection, GFP-positive cells were not detected in testicular sections using the applied detection method (Supplementary Figure S3). In contrast, the expression levels of endogenous SOX9 and CYP11A1 were markedly increased, suggesting that the beneficial effects of SVF are primarily mediated by paracrine mechanisms rather than by direct cell replacement.

It should be noted that the SVF in this study was derived from allogeneic GFP-transgenic donor rats and not from autologous sources. While allogeneic MSCs are considered hypoimmunogenic due to their low expression of major histocompatibility complex (MHC) molecules and absence of costimulatory molecules [38], the immunogenicity of uncultured SVF, which contains a heterogeneous mixture of cell types including immune cells, is less well characterized. Some studies have suggested that allogeneic adipose-derived cellular therapies may not elicit overt rejection in certain contexts, and SVF itself contains immunomodulatory populations such as regulatory T cells and M2-like macrophages that could potentially mitigate alloimmune responses [39]. Nevertheless, the potential for immune rejection cannot be excluded, particularly because SVF contains cells with antigen-presenting capacity [40,41]. The use of allogeneic SVF therefore represents a limitation when considering clinical translation, as autologous SVF would be preferred to eliminate immunogenicity concerns and avoid donor-to-donor variability. Future studies should directly compare allogeneic and autologous SVF in immunocompetent animal models to assess whether the therapeutic efficacy observed here is maintained and whether immune responses affect long-term outcomes.

A significant finding of this study is that, two weeks post-busulfan treatment, testosterone levels remained stable, and serum FSH, LH, and estradiol levels did not rise, despite evident testicular histopathological damage and diminished sperm parameters. This apparent dissociation between structural injury and endocrine function may be explained by the kinetics of the hypothalamic-pituitary-testicular (HPT) axis response. The initial injury from busulfan primarily affects rapidly dividing germ cells and spermatogonia, while Leydig cell endocrine function is only partially and progressively impaired [42,43]. Thus, the lack of a testosterone decline may reflect a “feedback lag period” before the HPT axis mounts a compensatory response. Our two-week endpoint may therefore represent an early phase in which the HPT axis has not yet initiated a full feedback response.

In contrast, SVF treatment significantly increased testosterone without altering FSH or LH levels. This dissociation between testosterone and gonadotropin regulation suggests that SVF may act directly on Leydig cells via a paracrine mechanism, apart from the classical LH-mediated pathway [44]. Several growth factors secreted by SVF, including HGF, VEGF, IGF-1 and PDGF, have been shown to stimulate Leydig cell steroidogenesis and improve testosterone production even in the absence of LH stimulation [45]. However, this interpretation is speculative because we did not directly assess Leydig cell steroidogenic activity (e.g., StAR expression, CYP11A1 enzyme activity, or LH receptor levels). Alternative explanations like effects on testosterone metabolism or clearance cannot be excluded and direct mechanistic studies are needed to confirm this hypothesis.

Besides the mechanistic uncertainty, it is also worth considering the timing of SVF administration. SVF was given immediately after busulfan exposure, which mainly assesses a protective or early intervention effect rather than a therapeutic effect on established injury. This design provides proof of concept that intravenous SVF can rapidly protect the testis from acute injury which is clinically relevant for conditions such as chemotherapy induced damage where early intervention may be feasible. However, delayed application would be more comparable to a true therapeutic setting, and future studies should address the efficacy of intravenous SVF when applied at later time points after busulfan exposure. Taken together, the hormonal profile observed at two weeks (busulfan without elevated FSH/LH and SVF increasing testosterone independently of gonadotropins) is consistent with a direct, rapid paracrine effect of SVF on Leydig cells. This mechanism may represent a significant benefit of cell-based therapies compared to hormone replacement, as it supports endogenous testosterone production without interfering with the HPT axis.

Our findings align with several studies employing cultured MSCs for testicular repair; however, those studies typically required prolonged observation periods (4–8 weeks) [7]. The only previous study on SVF in the busulfan model used intratesticular injection [26]. We build on that work by showing that intravenous delivery appears to be comparably effective. Compared with cultured MSCs, SVF offers several practical advantages, including rapid preparation (2–3 h without culture expansion), reduced manufacturing complexity, lower cost, and avoidance of culture-associated risks such as contamination, senescence, and genetic instability [46,47,48]. However, SVF is more heterogeneous than cultured MSCs, which may lead to batch-to-batch variability. Moreover, although cultured MSCs can be extensively characterized and standardized, SVF preparations require rigorous quality control. The choice to use either SVF or MSCs for a clinical application will depend on the clinical setting, taking into account the advantages of immediate, point-of-care availability versus the need for standardized, well-characterized cell products. For example, SVF may be relevant to clinical scenarios such as acute testicular injuries (e.g., torsion, trauma or chemical exposure) where an immediate cell-based intervention may be required.

Busulfan is known to causes oxidative stress, inflammation, apoptosis, and a disrupted stem cell niche, which are all common causes of spermatogenic impairment. These include testicular torsion, radiation exposure, varicocele, certain infections, and some genetic disorders. Importantly, these pathological mechanisms are not limited to rodents or to chemotherapy-induced models. Male reproductive injury associated with toxicological exposures has been documented across multiple species. For example, metals and metalloids have been associated with molecular and morphological changes in testes of dogs from animals living in polluted areas [49]. In bovine models, heavy metals such as lead and cadmium have been shown to have a negative effect on sperm motility and to cause alterations in the prooxidant/antioxidant balance in seminal plasma and spermatozoa [50]. The observations across species indicate that the pathogenic pathways induced by busulfan, including oxidative stress, inflammation, apoptosis and disturbance of the stem cell niche, are relevant to not only different etiologies but also different species. Consequently, SVF may provide advantages in various situations. Future studies should evaluate SVF in other models, such as ischemia–reperfusion, cryptorchidism, or autoimmune orchitis, to verify its broader relevance.

## 5. Research Limitations

Several limitations of this study should be acknowledged. The experiments were performed in a rodent model using busulfan as a single insult. While busulfan induces a reproducible and severe testicular damage that mimics many features of human spermatogenic impairment, it does not recapitulate all etiologies (e.g., genetic or chronic inflammatory conditions). Therefore, the translational relevance to other forms of spermatogenic impairment requires further validation. In particular, the only previous SVF study in this model used intratesticular injection [26]. We used intravenous delivery, and direct head-to-head comparisons of different routes are lacking. Another limitation is that we used healthy donor SVF rather than autologous SVF.

The follow-up period was only two weeks after busulfan injection, which is insufficient to cover a full spermatogenic cycle in rats (approximately 52 days). Consequently, we cannot determine whether the observed improvements represent true spermatogenic regeneration or reflect preservation of the pre-existing epididymal sperm reserve. To establish definitive spermatogenic restoration, future studies should incorporate longer observation periods covering multiple spermatogenic cycles, fertility assessment (e.g., mating trials and pregnancy rates), and molecular markers of germ-cell proliferation and differentiation (e.g., Ki-67 or PCNA).

We also note that reproductive competence (mating studies, pregnancy rates and/or offspring analyses) was not assessed in this study. Although we observed improvements in sperm parameters and testicular histology, whether these improvements translate to functional restoration of fertility remains to be seen. Future studies should incorporate fertility assessment to confirm functional relevance of these findings.

Also, the sample sizes were different for the different analyses (*n* = 3–6 per group) with smaller numbers being used for histological and immunohistochemical analyses. We recognize that the sample sizes are relatively small and variable, limiting the statistical power to detect subtle differences and may affect the reliability and generalizability of the findings. Future studies with larger and more consistent sample sizes are needed to confirm the observed effects. In addition, long-term safety and efficacy, including potential tumorigenicity or unwanted immune reactions, were not assessed. Future studies should address the above limitations by using autologous SVF, longer periods of observation, mechanistic dissection and finally large animal models and additional injury models prior to clinical trials.

Long-term safety and efficacy, including potential tumorigenicity or unwanted immune reactions, were not assessed. In addition, the optimal dose and frequency of SVF administration were not determined in this study. The batch-to-batch variability may affect the reproducibility, thus the standardization of the SVF preparation is still a challenge. In addition, although the use of healthy donor SVF in this study does not completely address potential immunogenicity issues related to allogeneic cell products, these issues should be systematically explored in future studies. Furthermore, the proposed paracrine and immunomodulatory mechanisms were not directly measured in this study; we did not quantify cytokines, growth factors, oxidative stress markers, or inflammatory mediators. Therefore, these mechanistic interpretations remain speculative and require direct validation in future studies. Future studies should address these limitations by using autologous SVF, longer observation periods, mechanistic dissection, and, ultimately, large animal models and additional injury models before clinical trials. findings are preliminary and require extensive further validation before any clinical translation.

## 6. Conclusions

In summary, a single intravenous administration of uncultured SVF swiftly ameliorates busulfan-induced testicular damage in rats, improving sperm quality, testicular structure, and Sertoli/Leydig/germ cell markers within two weeks. This is the first demonstration that systemic intravenous delivery of SVF is effective in this model, suggesting a less invasive alternative to the previously reported intratesticular route [26]. The observed effects are consistent with paracrine and immunomodulatory actions; however, these proposed mechanisms were not directly measured and therefore remain speculative. Given the short follow-up and absence of fertility data, our findings represent early protective evidence rather than definitive spermatogenic restoration. Longer-term studies with functional fertility assessment are needed to establish clinical relevance.

## Figures and Tables

**Table 1:** Cellular composition and marker genes used for cell type annotation in SVF identified by single-cell RNA sequencing (representative batch).

Cluster	The Number of Cells Within 8555 Total Rat SVF Cells	Percentage of Cells (%)	Marker Genes Used for Annotation
AD-SPCs	4071	47.59	Pdgfra, Has1
Macrophagocyte	3148	36.8	Cd163, Csf1r, Mrc1
T	313	3.66	Cd3d, Cd3g
Epithelial	67	0.78	Epcam, Klr18
Endo	303	3.54	Pecam1, Kdr, Flt1
Mast	363	4.24	Tpsb2, Fcer1a
ME	73	0.85	Hba-a1, Hbb-bs
B	16	0.19	Cd79a, Cd79b, Klrk1
NK	37	0.43	Klrk1, Klrb1b
Monocytes	164	1.92	Csf3r, Clec4e

AD-SPC: adipose stem and progenitor cells; Macrophage: macrophages; T: thymus-dependent lymphocytes; Epithelial: epithelial cells; Endothelial: endothelial cells; Mast: mast cells; Neutrophil: neutrophil cells; B: bone marrow-dependent lymphocytes. The percentage represents each subpopulation’s share of the 8555 cells that were subjected to single-cell RNA sequencing analysis.

## Data Availability

The single-cell RNA sequencing data generated in this study are available in the Gene Expression Omnibus (GEO) under accession number GSE339598. The data will be made publicly available upon publication. The other datasets analyzed during the current study are available from the corresponding author upon request.

## Supplementary Materials

The following supporting information can be downloaded at: https://www.mdpi.com/article/10.3390/biology15151312/s1, Figure S1: Flow cytometry gating strategy for SVF characterization.; Figure S2: Single-cell RNA-seq analysis of two additional independent SVF preparations (batches 2 and 3); Table S1: Cellular composition of two additional independent SVF preparations (batches 2 and 3) identified by single-cell RNA sequencing; Figure S3: GFP tracking of intravenously injected SVF cells.

## Abbreviations

The following abbreviations are used in this manuscript:

ADSC	adipose derived stem cell
ANOVA	analysis of variance
APC	allophycocyanin
CYP11A1	cholesterol side chain cleavage enzyme
DAB	3,3′ diaminobenzidine
DDX4	DEAD box helicase 4
DMEM	Dulbecco’s modified Eagle medium
DMSO	dimethyl sulfoxide
ELISA	enzyme linked immunosorbent assay
FSH	follicle stimulating hormone
GET	germinal epithelial thickness
GFP	green fluorescent protein
H&E	hematoxylin and eosin
HBSS	Hanks’ balanced salt solution
HPT	hypothalamic pituitary testicular
JMTBS	Johnsen mean tubular biopsy score
LH	luteinizing hormone
MSC	mesenchymal stem cell
PBS	phosphate-buffered saline
PE Cy7	phycoerythrin cyanine7
SD	Sprague Dawley
SOX9	SRY box transcription factor 9
STD	seminiferous tubule diameter
SVF	stromal vascular fraction
TEM	transmission electron microscopy
t SNE	t distributed stochastic neighbor embedding

## References

[1] Meistrich M.L. (2013). Effects of chemotherapy and radiotherapy on spermatogenesis in humans. Fertil. Steril..

[2] Howell S.J., Shalet S.M. (2005). Spermatogenesis after cancer treatment: Damage and recovery. J. Natl. Cancer Inst. Monogr..

[3] Agarwal A., Baskaran S., Parekh N., Cho C.L., Henkel R., Vij S., Arafa M., Panner Selvam M.K., Shah R. (2021). Male infertility. Lancet.

[4] Bucci L.R., Meistrich M.L. (1987). Effects of busulfan on murine spermatogenesis: Cytotoxicity, sterility, sperm abnormalities, and dominant lethal mutations. Mutat. Res..

[5] Sasso-Cerri E., Oliveira B., de Santi F., Beltrame F.L., Caneguim B.H., Cerri P.S. (2017). The antineoplastic busulphan impairs peritubular and leydig cells, and vitamin b(12) stimulates spermatogonia proliferation and prevents busulphan-induced germ cell death. Biomed. Pharmacother..

[6] Ehmcke J., Wistuba J., Schlatt S. (2006). Spermatogonial stem cells: Questions, models and perspectives. Hum. Reprod. Update.

[7] El-Wafaey D.I., Talaat A., El-Malkey N.F., Abdelhamid A.M., Ibrahim F., Zaazaa A.M., Faruk E. (2025). Quercetin attenuates busulfan-induced testicular and epididymal toxicity via modulation of oxidative stress, inflammatory cytokines, and apoptotic markers in rats. Eur. J. Pharmacol..

[8] Goossens E., Jahnukainen K., Mitchell R.T., van Pelt A., Pennings G., Rives N., Poels J., Wyns C., Lane S., Rodriguez-Wallberg K.A. (2020). Fertility preservation in boys: Recent developments and new insights (†). Hum. Reprod. Open.

[9] Irani D., Mehrabani D., Karimi-Busheri F. (2024). Mesenchymal stem cells in regenerative medicine, possible applications in the restoration of spermatogenesis: A review. Cell J..

[10] Huang G., Quan L., Li Q., Zhou X., Han M., Peng F., Gong Y. (2024). Umbilical cord-derived mesenchymal stem cells improve ornidazole-induced asthenozoospermia in rats via activation of the AKT/mTOR pathway. Int. J. Endocrinol..

[11] Evangelista N.N., Shafira I.D., Sylviana N., Rezano A. (2026). Adipose-derived mesenchymal stem cell secretome promotes testicular regeneration following chemically induced injury: A review of preclinical studies. Arch. Ital. Urol. Androl..

[12] Abdelbaset S., Mohamed Sob M.A., Mutawa G., El-Dein M.A., Abou-El-Naga A.M. (2024). Therapeutic potential of different injection methods for bone marrow mesenchymal stem cell transplantation in buslfan-induced male rat infertility. J. Stem Cells Regen. Med..

[13] Amirjannati N., Barfar M., Pournourali M., Khalili N., Tajik P., Shams R., Jahandideh A., Goli L.B., Hashemian S.J., Shirazi A. (2026). The secretome from human amniotic membrane-derived mesenchymal stem cells alleviate oxidative damage in bovine sperm. Reprod. Biol..

[14] Trounson A., McDonald C. (2015). Stem cell therapies in clinical trials: Progress and challenges. Cell Stem Cell.

[15] Prisciandaro M., Santinelli E., Tomarchio V., Tafuri M.A., Bonchi C., Palazzo G., Nobile C., Marinucci A., Mele M., Annibali O. (2024). Stem cells collection and mobilization in adult autologous/allogeneic transplantation: Critical points and future challenges. Cells.

[16] Thong C.Y.E.N., Yap Y.H.Y. (2026). Stem cells as emerging regenerative approaches for post-traumatic stress disorder: Mechanisms and translational challenges. Brain Res..

[17] Hurd R.E. (1992). Ethical issues surrounding the transplantation of human fetal tissues. Clin. Res..

[18] Sanders L.M., Giudice L., Raffin T.A. (1993). Ethics of fetal tissue transplantation. West. J. Med..

[19] Nonnarath C., Serratrice N. (2026). Safety profile of autologous adipose-derived stromal vascular fraction in clinical use: An exhaustive literature review. Stem Cell Res. Ther..

[20] Chen X., Li T., Yang F., Chen Y., Liu Y., Ding L., Li J., Zhou H., Yuan Q., Wu W. (2026). Combined stromal vascular fraction and HGF-functionalized self-assembling peptide hydrogel improves intracerebral hemorrhage repair in rats. Gels.

[21] Aronowitz J.A., Lockhart R.A., Hakakian C.S., Hicok K.C. (2015). Clinical safety of stromal vascular fraction separation at the point of care. Ann. Plast. Surg..

[22] Cohen S.R., Wesson J., Canikyan S., Tiryaki T. (2025). Potential medical and surgical applications of stromal vascular fraction and adipose-derived stem cells: A narrative review. HSS J..

[23] Gareev I., Beylerli O., Ilyasova T., Ahmad A., Shi H., Chekhonin V. (2024). Therapeutic application of adipose-derived stromal vascular fraction in myocardial infarction. iScience.

[24] Prescher H., Froimson J.R., Hanson S.E. (2023). Deconstructing fat to reverse radiation induced soft tissue fibrosis. Bioengineering.

[25] Rehman J., Traktuev D., Li J., Merfeld-Clauss S., Temm-Grove C.J., Bovenkerk J.E., Pell C.L., Johnstone B.H., Considine R.V., March K.L. (2004). Secretion of angiogenic and antiapoptotic factors by human adipose stromal cells. Circulation.

[26] Hekimoglu E.R., Esrefoglu M., Karakaya Cimen F.B., Elibol B., Dedeakayogullari H., Pasin Ö. (2024). Beneficial effects of adipose-derived stromal vascular fraction on testicular injury caused by busulfan. Drug Chem. Toxicol..

[27] Zhou L., Song K., Xu L., Zhao F., Tian H., Zhou C., Xu Z., Ge Y., Wu R., Jia R. (2019). Protective effects of uncultured adipose-derived stromal vascular fraction on testicular injury induced by torsion-detorsion in rats. Stem Cells Transl. Med..

[28] Amin S.E., Farghaly E.F., Abdelmonsif D.A., Zaki E.I., Omar W. (2026). The potential ameliorative effect of curcumin nanoemulsion on busulfan-induced testicular toxicity in adult rat model. Reprod. Toxicol..

[29] Askar E.M., Abdelmegid A.M., Elshal L.M., Shaheen M.A. (2024). Effect of platelet rich plasma versus melatonin on testicular injury induced by busulfan in adult albino rats: A histological and immunohistochemical study. Ultrastruct. Pathol..

[30] Pu R., Liu J., Zhang A., Yang J., Zhang W., Long X., Ren X., Hua H., Shi D., Zhang W. (2023). Modeling methods for busulfan-induced oligospermia and asthenozoospermia in mice: A systematic review and meta-analysis. J. Assist. Reprod. Genet..

[31] Khanlarkhani N., Pasbakhsh P., Mortezaee K., Naji M., Amidi F., Najafi A., Sobhani A., Zendedel A. (2016). Effect of human recombinant granulocyte colony-stimulating factor on rat busulfan-induced testis injury. J. Mol. Histol..

[32] Bourguiba S., Genissel C., Lambard S., Bouraïma H., Carreau S. (2003). Regulation of aromatase gene expression in leydig cells and germ cells. J. Steroid Biochem. Mol. Biol..

[33] Liu Z.J., Liu Y.H., Huang S.Y., Wu C.L., Zang Z.J. (2023). Effects of perfluorododecanoic acid on testicular function in mice. Toxicol. Res..

[34] Chun S.Y., Lim J.O., Lee E.H., Han M.H., Ha Y.S., Lee J.N., Kim B.S., Park M.J., Yeo M., Jung B. (2019). Preparation and characterization of human adipose tissue-derived extracellular matrix, growth factors, and stem cells: A concise review. Tissue Eng. Regen. Med..

[35] Hakovirta H., Yan W., Kaleva M., Zhang F., Vänttinen K., Morris P.L., Söder M., Parvinen M., Toppari J. (1999). Function of stem cell factor as a survival factor of spermatogonia and localization of messenger ribonucleic acid in the rat seminiferous epithelium. Endocrinology.

[36] LeCouter J., Lin R., Tejada M., Frantz G., Peale F., Hillan K.J., Ferrara N. (2003). The endocrine-gland-derived VEGF homologue bv8 promotes angiogenesis in the testis: Localization of bv8 receptors to endothelial cells. Proc. Natl. Acad. Sci. USA.

[37] Fujita M., Matsumoto T., Hayashi S., Hashimoto S., Nakano N., Maeda T., Kuroda Y., Takashima Y., Kikuchi K., Anjiki K. (2022). Paracrine effect of the stromal vascular fraction containing m2 macrophages on human chondrocytes through the smad2/3 signaling pathway. J. Cell. Physiol..

[38] Keshavarz Shahbaz S., Mansourabadi A.H., Jafari D. (2022). Genetically engineered mesenchymal stromal cells as a new trend for treatment of severe acute graft-versus-host disease. Clin. Exp. Immunol..

[39] Locatelli M., Boehncke W.H.N., Pastor D., Villard J., Brembilla N.C., Preynat-Seauve O. (2026). Gelatin sponge-embedded adipose-derived stromal cells enable allogeneic application for revascularization of ischemic wounds. Int. J. Mol. Sci..

[40] Chang S.H., Kim H.J., Park C.G. (2020). Allogeneic ADSCs induce the production of alloreactive memory-CD8 t cells through HLA-ABC antigens. Cells.

[41] Lee J.W., Park B.C., Jang N.Y., Lee S., Cho Y.K., Sharma P., Byun S.W., Jeon K., Jeon Y.H., Park U. (2022). Inducing ectopic t cell clusters using stromal vascular fraction spheroid-based immunotherapy to enhance anti-tumor immunity. Adv. Sci..

[42] Gomes W.R., Hall R.W., Jain S.K., Boots L.R. (1973). Serum gonadotropin and testosterone levels during loss and recovery of spermatogenesis in rats. Endocrinology.

[43] Debeljuk L., Arimura A., Schally A.V. (1973). Pituitary and serum FSH and LH levels after massive and selective depletion of the germinal epithelium in the rat testis. Endocrinology.

[44] Saez J.M. (1994). Leydig cells: Endocrine, paracrine, and autocrine regulation. Endocr. Rev..

[45] Zachow R., Uzumcu M. (2007). The hepatocyte growth factor system as a regulator of female and male gonadal function. J. Endocrinol..

[46] Lepperdinger G., Brunauer R., Jamnig A., Laschober G., Kassem M. (2008). Controversial issue: Is it safe to employ mesenchymal stem cells in cell-based therapies?. Exp. Gerontol..

[47] Prockop D.J., Brenner M., Fibbe W.E., Horwitz E., Le Blanc K., Phinney D.G., Simmons P.J., Sensebe L., Keating A. (2010). Defining the risks of mesenchymal stromal cell therapy. Cytotherapy.

[48] Simão V.A., So R.C.P., Leme J., Oliveira R.G.D., Leite G.A.A., Chuffa L.G.D.A., Tonso A., Ribeiro-Paes J.O.T. (2026). Genotoxicity integration into bioprocess optimization reveals progressive DNA damage during bioreactor expansion of adipose-derived stem cells. Int. J. Mol. Sci..

[49] Riccio L., De Falco M., Trifuoggi M., Cucolo C., Conte F., Chianese T., Ambrosio N., Spada A., Di Napoli E., Paciello O. (2026). “Testicular concentrations of metals and metalloids in dogs from the ‘land of fires’ (campania region, italy): A preliminary morphological, chemical and molecular study”. Environ. Toxicol. Pharmacol..

[50] Tvrdá E., Kňažická Z., Lukáčová J., Schneidgenová M., Goc Z., Greń A., Szabó C., Massányi P., Lukáč N. (2013). The impact of lead and cadmium on selected motility, prooxidant and antioxidant parameters of bovine seminal plasma and spermatozoa. J. Environ. Sci. Health Part A.

