# Supplementary material for: Intravenous Administration of Stromal Vascular Fraction Attenuates Early Busulfan-Induced Testicular Injury in Rats: An Alternative Cell-Based Approach for Spermatogenic Impairment
Source: Biology (Basel). 2026 Aug 5;15(15):1312. doi: 10.3390/biology15151312 (PMC13465997; doi:10.3390/biology15151312)
Supplement: Supplementary file 1 [file biology-15-01312-s001.zip › biology-4407041-supplementary.pdf]

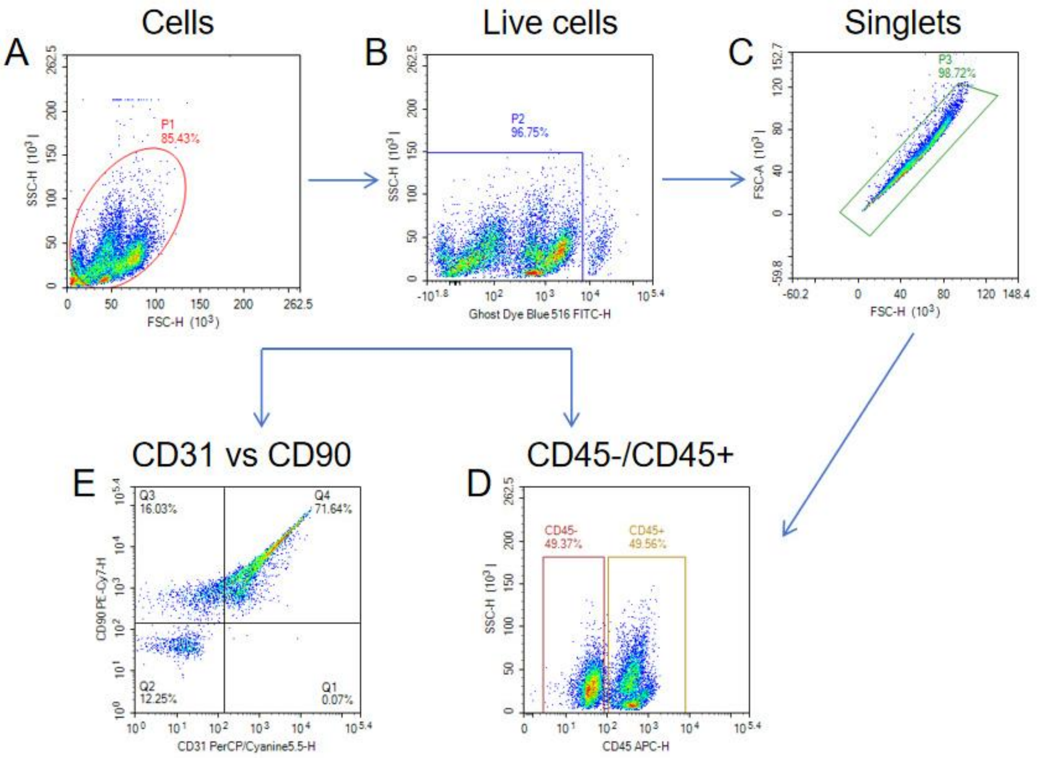

Supplementary Figure S1. Flow cytometry gating strategy for SVF characterization. Representative flow cytometry plots showing the sequential gating strategy used to characterize the cellular composition of freshly isolated SVF. **(A)** Forward scatter-area (FSC-A) versus side scatter-area (SSC-A) was used to exclude debris and select the main cell population (P1, 85.43% of total events). **(B)** Ghost Dye Blue 516 staining versus SSC-A was used to identify viable cells (P3, 98.72% of P1). **(C)** FSC-A versus FSC-height (FSC-H) was used to exclude doublets and select single cells (P2, 96.75% of P3). **(D)** The viable single-cell population was further gated on CD45 expression to distinguish $CD45^+$ hematopoietic cells (49.56%) and $CD45^-$ non-hematopoietic cells (49.37%). **(E)** Among the $CD45^-$ population, cells were analyzed for CD31 (endothelial marker) and CD90 (MSC marker) expression: $CD31^+CD90^-$ (16.03%) and $CD31^-CD90^+$ (71.64%). Percentages in panels (A–C) indicate the proportion of events relative to the preceding gate; percentages in panels (D–E) indicate the proportion of cells within the indicated parent gate.

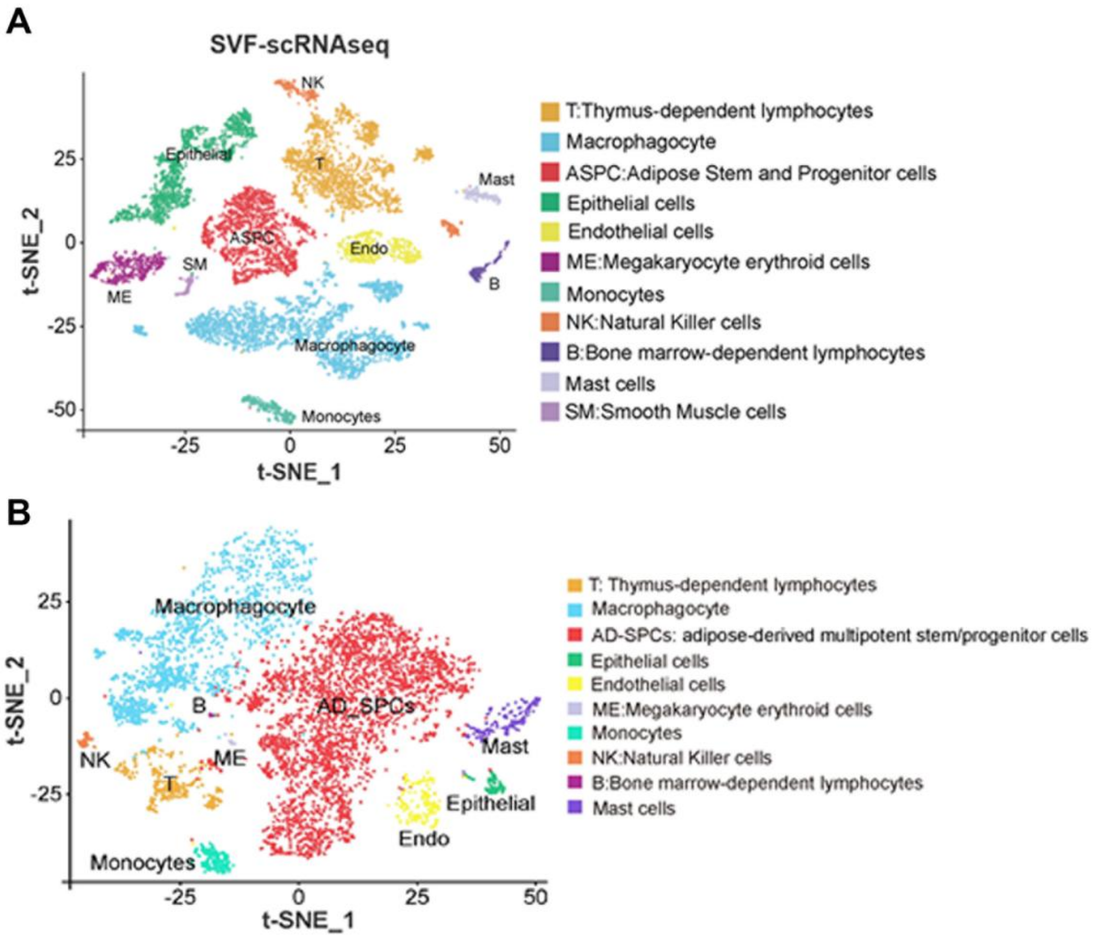

Supplementary Figure S2. Single-cell RNA-seq analysis of two additional independent SVF preparations (batches 2 and 3). (A–B) t-SNE plots showing the cellular heterogeneity of freshly isolated SVF cells from batch 2 (A) and batch 3 (B), respectively. Each dot represents a single cell, and cells are colored according to their assigned cell type annotation based on canonical marker gene expression. The same 10 major cell populations identified in the representative preparation (Figure 1B) including macrophages, adipose stem and progenitor cells (AD-SPCs), T cells, endothelial cells, and other minor populations, were consistently observed in both additional batches, suggesting the reproducibility of the SVF cellular composition across biological replicates.

Supplementary Table S1: Cellular composition of two additional independent SVF preparations (batches 2 and 3) identified by single-cell RNA sequencing

**cluster**	**batch 2: Total cell count: 8,546; number and percentage (%) of each cell type (Published)**	**batch 3: Total cell count: 6095; number and percentage (%) of each cell type**
AD-SPC	1544（16.74%）	3325　(54.55%)

Macrophagocyte	2583(28.01%)	1506 (24.71%)
T	1845(20.01%)	500 (8.2%)
Mast	162（1.76%）	177 (2.9%)
Endo	545（5.91%）	193 (3.17%)
Monocyte	257(2.79%)	199 (3.26%)
Epithelial	1263(13.7%)	106 (1.74%)
NK	287(3.11%)	65 (1.07%)
ME	470(5.1%)	8 (0.13%)
B	167(1.81%)	16 (0.26%)

AD-SPC: adipose stem and progenitor cells; Endo: endothelial cells; ME: megakaryocyte-erythroid progenitor cells; NK: natural killer cells; T: thymus-dependent lymphocytes; B: bone marrow-dependent lymphocytes; Macrophagocytes: macrophages; Epithelial: epithelial cells; Endothelial: endothelial cells; SM: smooth muscle cells; Mast: mast cells. The percentage represents each subpopulation's share of the total cells that were subjected to single-cell RNA sequencing analysis.

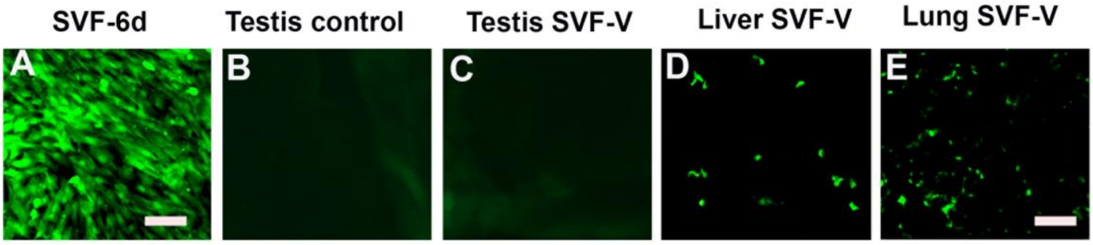

Supplementary Figure S3. GFP tracking of intravenously injected SVF cells. Representative fluorescence microscopy images of tissue sections from rats that received intravenous injection of GFP-transgenic SVF cells (SVF-V group), harvested at two weeks post-injection. GFP-positive cells are shown in green. **(A)** GFP-positive SVF cells cultured for 6 days in vitro (positive control). **(B)** Testicular tissue from a normal (non-transgenic) SD rat (negative control). **(C)** Testicular tissue from an SVF-treated rat; no GFP-positive cells were detected. **(D)** Liver tissue from an SVF-treated rat, showing sparse GFP-positive cells. **(E)** Lung tissue from an SVF-treated rat, showing abundant GFP-positive cells. The GFP-positive cells in the lung (and to a lesser extent in the liver) confirms successful cell delivery, while the absence of GFP signal in the testis indicates that significant engraftment did not occur in the target organ. Scale bar in A = 50 μm. Scale bar in E = 100 μm.
